# Deficiency of *Gpr1* improves steroid hormone abnormality in hyperandrogenized mice

**DOI:** 10.1186/s12958-018-0363-9

**Published:** 2018-05-24

**Authors:** Ya-Li Yang, Li-Feng Sun, Yan Yu, Tian-Xia Xiao, Bao-Bei Wang, Pei-Gen Ren, Hui-Ru Tang, Jian V. Zhang

**Affiliations:** 1 Research Laboratory for Reproductive Health, Shenzhen Institutes of Advanced Technology, Chinese Academy of Sciences, Shenzhen, 518055 China; 2 University of Chinese Academy of Sciences, Beijing, 100049 China; 3 Baoan Maternal and Child Health Care Hospital, Shenzhen, 518101 China; 4 Peking University Shenzhen Hospital, Shenzhen, 518035 China

**Keywords:** GPR1, Hyperandrogenism, Steroidogenesis, Estradiol

## Abstract

**Background:**

Polycystic ovary syndrome (PCOS) is a complex genetic disease with multifarious phenotypes. Many researches use dehydroepiandrosterone (DHEA) to induce PCOS in pubertal mouse models. The aim of this study was to investigate the role of GPR1 in dehydroepiandrosterone (DHEA)-induced hyperandrogenized mice.

**Methods:**

Prepubertal C57BL/6 mice (25 days of age) and Gpr1-deficient mice were each divided into two groups and injected daily with sesame oil with or without DHEA (6 mg/100 g) for 21 consecutive days. Hematoxylin and eosin (H&E) staining was performed to determine the characteristics of the DHEA-treated ovaries. Real-time PCR was used to examine steroid synthesis enzymes gene expression. Granulosa cell was cultured to explore the mechanism of DHEA-induced, GPR1–mediated estradiol secretion.

**Results:**

DHEA treatment induced some aspects of PCOS in wild-type mice, such as increased body weight, elevated serum testosterone, increased number of small, cystic, atretic follicles, and absence of corpus luteum in ovaries. However, *Gpr1* deficiency significantly attenuated the DHEA-induced weight gain and ovarian phenotype, improving steroidogenesis in ovaries and estradiol synthesis in cultured granulosa cells, partially through mTOR signaling.

**Conclusions:**

In conclusion, *Gpr1* deficiency leads to the improvement of steroid synthesis in mice hyperandrogenized with DHEA, indicating that GPR1 may be a therapeutic target for DHEA-induced hyperandrogenism.

**Electronic supplementary material:**

The online version of this article (10.1186/s12958-018-0363-9) contains supplementary material, which is available to authorized users.

## Background

Dehydroepiandrosterone (DHEA) is the most abundant steroid in human blood circulation. Synthesized mainly in the adrenal zona reticularis [1], it is an important precursor in follicular steroidogenesis and plays roles in protecting the central nervous system, preventing neurodegenerative diseases [2], improving depression [3], regulating and stabilizing inflammation and cellular immune functions [4], improving blood lipid metabolism [5], preventing osteoporosis [6], and protecting the cardiovascular system [7]. Since DHEA can be converted to androgens and estrogens, high levels of DHEA have been found to affect ovarian folliculogenesis [8] and lipid metabolism [9].

DHEA is generally increased in women with polycystic ovary syndrome (PCOS) [10]. PCOS is the most common and complex endocrine disorder affecting women of reproductive age and is characterized by hyperandrogenemia, menstrual irregularities, anovulation, infertility, obesity and hirsutism [11]. Women with PCOS are also at high risk of developing diabetes, insulin resistance, metabolic dysfunction, glucose intolerance, and cardiovascular disease [12].

Owing to the heterogeneous presentations of PCOS, it is difficult to develop an animal model that could mimic all the phenotypes observed in clinical. To date, PCOS-like animal model could be established by several approaches. Androgen-induced rodent PCOS models, based on DHEA, DHT or testosterone- present hyperandrogenemia, anovulation, cystic ovaries and development of impaired insulin/glucose metabolism [13]. Estrogen-induced rodent PCOS models, using estradiol benzoate, E2 and E2 valerate- can result in polycystic ovaries and anovulation but lack of metabolic features associated with human PCOS [14]. Aromatase inhibitor-letrozole-induced rodent PCOS models can cause ovulatory failure and polycystic ovaries [15]. And some chemicals, such as RU486, a progesterone receptor antagonist which inhibits the development and maturation of follicles, can result in delayed ovulation and induction of hyperprolactinemia like PCOS [16]. Besides, environmental influences, such as high fat diet [17], excess soy [18] and light, may also induce PCOS characteristics especially related metabolic co-morbidities. In addition, Transgenic modifications, such as the overexpression of NGF (nerve growth factor) in the ovary [19] or the overexpression of PAI-1(plasminogen activator inhibitor-1) in the ovary could cause PCOS in mice. Comparatively, among these approaches above, the most common PCOS models are based on hyperandrogenism induced pre−/post-natal stage or adulthood, since it is generally believed that increased androgen level is the main cause of PCOS [20].

Many researches use DHEA to induce PCOS in pubertal mouse models. Mice treated with DHEA are infertile, and their ovaries contain more cystic and atretic follicles and lack corpus luteum. Serum hormone levels, metabolic parameters, and inflammatory signals are also changed after DHEA treatment [21–23]. Whether DHEA treatment in rodents is an ideal approach to mimic human PCOS is still uncertain; however, the results of many studies indicate that postnatal treatment of mice with DHEA induces at least some aspects of PCOS, such as bodyweight gain, elevated serum insulin and glucose and more atretic follicles [20].

Chemerin is both a chemokine and an adipokine and plays important roles in immune responses, inflammation, adipogenesis, and carbohydrate metabolism [24]. Clinical data have shown that serum Chemerin levels are increased in women with PCOS and decrease after metformin treatment [25], indicating that Chemerin may be involved in the development of the metabolic disorders associated with PCOS. Our previous research has shown that Chemerin inhibits gonad hormone secretion from testes and ovaries [26].

So far, three endogenous receptors for Chemerin have been identified: GPR1, CMKLR1, and CCRL2 [24]. Among these, CCRL2 has not been shown to transduce the Chemerin signal but may increase local Chemerin concentration and affect inflammation via CMKLR1 [27]. CMKLR1 is the most well-characterized Chemerin receptor, and most of the biological functions of Chemerin have been shown to depend on CMKLR1 activation [28]. Our recent study showed that CMKLR1 gene deletion attenuates the effects of chronic DHT treatment on ovarian function in experimental PCOS [29]. Although Chemerin is the only known ligand for GPR1, few functions have been suggested for GPR1. It has been reported that GPR1 is expressed in metabolically active tissues and plays a functional role in glucose homoeostasis in obesity [30]. A new study from our lab shows that Chemerin and GPR1 are expressed in the mouse ovary during the estrous cycle and that Chemerin/GPR1 signaling could suppress hCG-induced progesterone synthesis and secretion during follicle development, partly through PI3K [31].

Whether GPR1 plays a regulatory role in hormone secretion and metabolism in hyperandrogenic mice is still unknown; there have been no previous reports on the relationship between the Chemerin receptor GPR1 and hyperandrogenism. Here we compare wild-type and *Gpr1* knockout mice in our model of DHEA-induced hyperandrogenism to explore the role of GPR1 in bodyweight, steroidogenesis and metabolism.

## Methods

### Animals

*Gpr1* knockout mice in the C57BL/6 J background were provided by Deltagen Ltd. and the Zabel lab, the validations for Gpr1 knockout mice were shown in an (Additional file 1: Figure S1). C57BL/6 J wild type female mice were obtained from the Laboratory Animal Center, Institutes of Biomedicine and Health, Chinese Academy of Sciences, China. Mice were maintained under standard housing conditions with controlled temperature and humidity, a 12-h light-dark cycle, and ad libitum access to food and water. All procedures related to animal use were approved by the Committee on the Use of Live Animals for Teaching and Research, Shenzhen Institutes of Advanced Technology, Chinese Academy of Sciences.

### Establishment of DHEA-induced hyperandrogenic mice

Pre-pubertal C57BL/6 mice (25 days of age) and *Gpr1* knockout mice were each divided into two groups (control and DHEA; 6 per group) and injected subcutaneously daily with sesame oil or sesame oil with DHEA (6 mg/100 g) for 21 consecutive days (Hubei Danjiangkou Kaitai Hormone CO., LTD). Body weights were determined every 3 days during treatment. At the end of the treatment period, mice were anesthetized and blood samples were collected by removal of the eyeballs. Mice were then euthanized by cervical dislocation, and tissues were collected. Aliquots of serum samples were sent to the Beijing North Institute of Biological Technology (Beijing, China) to measure levels of estradiol, progesterone, and testosterone. Separate aliquots of serum were sent to Shenzhen SHAHE Hospital (Shenzhen, China) to measure serum lipids. Hypothalamus and pituitary glands were isolated and stored in RNAiso Plus (Takara Bio) at − 80 °C for subsequent total RNA isolation. Ovaries and fat tissues were either put in RNAiso Plus for total RNA isolation or fixed in Bouin’s solution and embedded in paraffin for histological examination.

### RNA analysis by quantitative PCR

Total RNA from tissues and cells was extracted using RNAiso Plus reagent. cDNAs were synthesized using ReverTra Ace qPCR RT Kit (Toyobo, Osaka, Japan) for subsequent real-time quantitative PCR analysis. The PCR reaction mixtures contained 10 ul SYBR® Premix Ex Taq II (Toyobo), 1 ul (0.5 pmole/μl) of each primer, 1 ul template cDNA, and 7 ul DNase-free water to a final volume of 20 ul. The conditions used for real-time quantitative PCR were as follows: 95 °C for 10 s, followed by 45 cycles at 95 °C for 5 s, 60°Cfor 30 s, and 72 °C for 30 s. The reaction was completed with a dissociation step for melting point analysis from 50 °C to 95 °C (in increments of 0.5 °C for 10 s each). All reactions were run in duplicate. B-actin was used as the reference gene. The RNA levels were calculated by the 2 − ΔCT method, where CT was the cycle threshold. Primer pairs specific for each gene were designed on the basis of the published sequences and are presented in Table 1.

**Table 1:** Primer pairs specific for each gene used in the study

Name	Fw Primer Sequence (5′-3′)	Rv Primer Sequence (5′-3′)	Target Sequence
*GnRH*	gtggatcaaatggcagaaccc	gggccagtgcatctacatct	NM_008145.3
*AR*	tccaagacctatcgaggagcg	gtgggcttgaggagaaccat	NM_013476.4
*ERA*	cccgccttctacaggtctaat	ctttctcgttactgctggacag	NM_007956.5
*LH*	tggccgcagagaatgagttc	ctcggaccatgctaggacagtag	NM_008497.2
*FSH*	ggagagcaatctgctgccata	gcagaaacggcactcttcct	NM_008045.3
*GH*	ctacaaagagttcgagcgtgcctac	caattccatgtcggttctctgc	NM_008117.3
*PRL*	agggagttgagaagataattagccag	aagaggagaccaattgcaccca	NM_011164.2
*UCP1*	actgccacacctccagtcatt	ctttgcctcactcaggattgg	NM_009463.3
*StAR*	ccgggtggatgggtcaa	cacctctccctgctggatgta	NM_011485.4
*P450scc*	ccatcagatgcagagtttccaa	tgagaagagtatcgacgcatcct	NM_019779.3
*3B-HSD*	ggaggcctgtgttcaagcaa	ggccctgcaacatcaactg	NM_008293.4
*17B-HSD*	ttgtttgggccgctagaag	cacccacagcgttcaattca	NM_010475.1
*Aromatase*	gcaatcctgaaggagatcca	gccgtcaattacgtcatcct	NM_007810.3
*Hsd17b7*	cctgtgctcagtccgttttt	ccaaggccctgaattcaata	NM_010476.3

Supplement: Internal Reference βactin Fw,gtgacgttgacatccgtaaaga Rv,gccggactcatcgtactccTarget,NM_007393.5

### Histology

Fixed ovaries and fat tissues were embedded in paraffin, and 5 μm sections were cut and stained with hematoxylin and eosin (H&E). The number of cystic follicles (with granulosa cells degenerated) and corpus luteum were counted in more than six slides. The diameters of adipocytes were determined using Image- Pro Plus 6.0.

### Granulosa cell culture

21-day-old female C57BL/6 mice were injected i.p. with 5 IU of PMSG (ProSpec) to initiate follicular development. To stimulate ovulation and luteinization, mice were injected 48 h after PMSG injection with 5 IU of hCG (Sigma) and then collected 7–12 h later. Mice were then euthanized by cervical dislocation, ovaries were removed, and fat and mesangial tissue around ovaries were removed using a 1 mL syringe with needle. Granulosa cells were obtained from the ovarian tissue by follicular puncture, and placed in PBS. The cell suspension was filtered through a 70 um mesh sieve three times to remove oocytes and centrifuged at 1000×g for 5 min. The pellet was resuspended in serum-free DMEM F12 containing 3% BSA and insulin-transferrin-sodium selenite (ITS, Sigma). For in vitro analysis, granulosa cells were co-cultured with 10^− 3^ nM of the mTOR inhibitor rapamycin (Selleck). For groups receiving antibody treatment, 0.5 μg/mL mouse anti-GPR1 antibody (Zabel laboratory) was added. Control groups received 0.5 μg/mL rat IgG (Abcam). Plates were incubated at 37 °C, 5% CO2, and cells and media were collected after 24 h. A CCK-8 assay (Beyotime, China) was employed to quantitatively evaluate cell proliferation. Media were analyzed for hormone levels by the Beijing North Biotechnology Research Institute, and cells were placed in RNAiso Plus and stored at − 80 °C for qPCR.

### Hormone measurements

Serum testosterone, estradiol, and progesterone levels were measured using commercial iodine [125I] RIA Kits (Beijing North Biotechnology Research Institute). Serum HDL (High Density Lipoprotein), LDL (Low Density Lipoprotein), CHO (Cholesterol), and TG (Triglyceride) levels were measured using the cobas c311 automatic biochemical analyzer (Roche, Shenzhen SHAHE Hospital). The intra- and inter-assay errors among all assays were less than 10 and 15%, respectively.

### Statistical analysis

All data are presented as means ± SEM. Statistical analysis of differences between two groups was performed using the Student’s t-test; a *P* value < 0.05 was considered statistically significant. All analyses were performed using GraphPad Prism software (GraphPad Software Inc., San Diego, CA, USA).

## Results

### Body weight gain and serum lipid and hormone levels

**Fig. 1:**
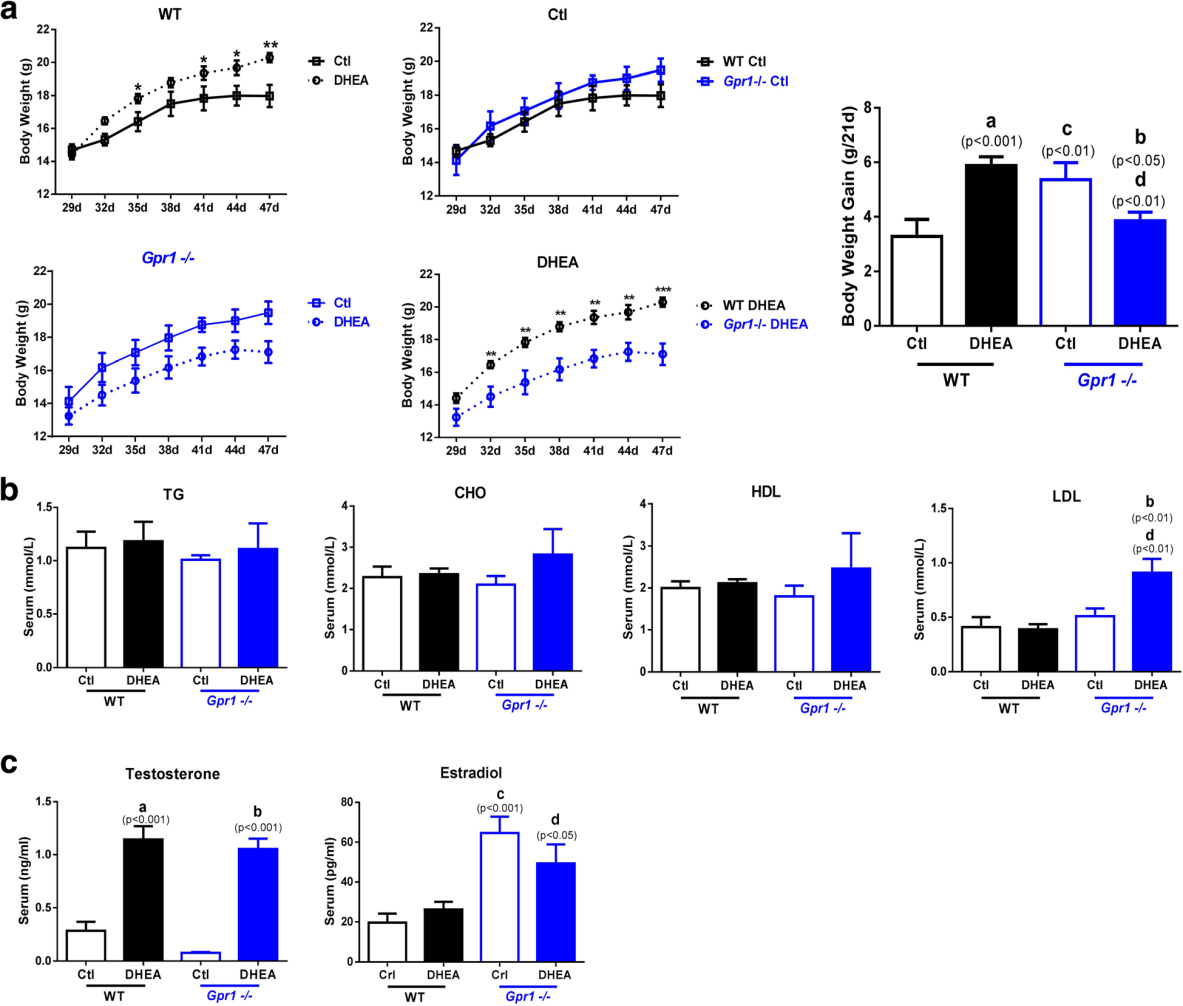
Body weight and serum lipid and hormone levels in control and DHEA-treated wild type and *Gpr1*-null mice (n > 6). A Body weight and body weight gain in control (Ctl) and DHEA-treated wild type and *Gpr1*-null mice. B Serum HDL, LDL, CHO, and TG levels in CTL and DHEA-treated wild type and *Gpr1*-null mice. C Serum testosterone and estradiol levels in CTL and DHEA-treated wild type and *Gpr1*-null mice. Data were analyzed using an unpaired Student’s t test. (a) *p*, DHEA-treated vs. control wild type mice; (b) *p*, DHEA-treated vs. control *Gpr1*-null mice; (c) *p,* control *Gpr1*-null mice vs. control wild type mice; (d) *p*, DHEA-treated *Gpr1* null mice vs. DHEA-treated wild type mice

Body weight gains were significantly greater in DHEA-treated than in control wild type female mice. In *Gpr1*-deficient mice, body weight gains were significantly greater than in wild type mice, but body weight gains in DHEA-treated *Gpr1*-deficient mice were less than those in both DHEA-treated wild type mice and untreated *Gpr1*-deficient mice (Fig. 1A), indicating that GPR1 may play a role in weight change and that *Gpr1* deficiency suppresses DHEA-induced bodyweight gain.

Serum lipid and hormone levels were measured in control and DHEA-treated wild type and *Gpr1*-deficient mice. The serum lipid levels of wild type mice were not affected by DHEA, while in *Gpr1*-deficient mice, LDL levels were significantly elevated after DHEA treatment. HDL, CHO, and TG levels were not significantly different among the four groups (Fig. 1B).

Testosterone levels were significantly greater in both wild type and *Gpr1* knockout mice after DHEA treatment than they were in the control mice, confirming proper establishment of the in vivo hyperandrogenism model. *Gpr1* deficiency significantly elevated estradiol levels in both DHEA-treated and control mice (Fig. 1C).

### Relative mRNA expression for steroid receptors and hormones in the hypothalamus and pituitary gland

To determine the effect of hyperandrogenism on the hypothalamic-pituitary-gonadal axis, we measured the levels of mRNAs encoding steroid receptors and *GnRH* (Gonadotropin Releasing Hormone) in the hypothalamus and those encoding several hormones in the pituitary glands of the four experimental groups.

**Fig. 2:**
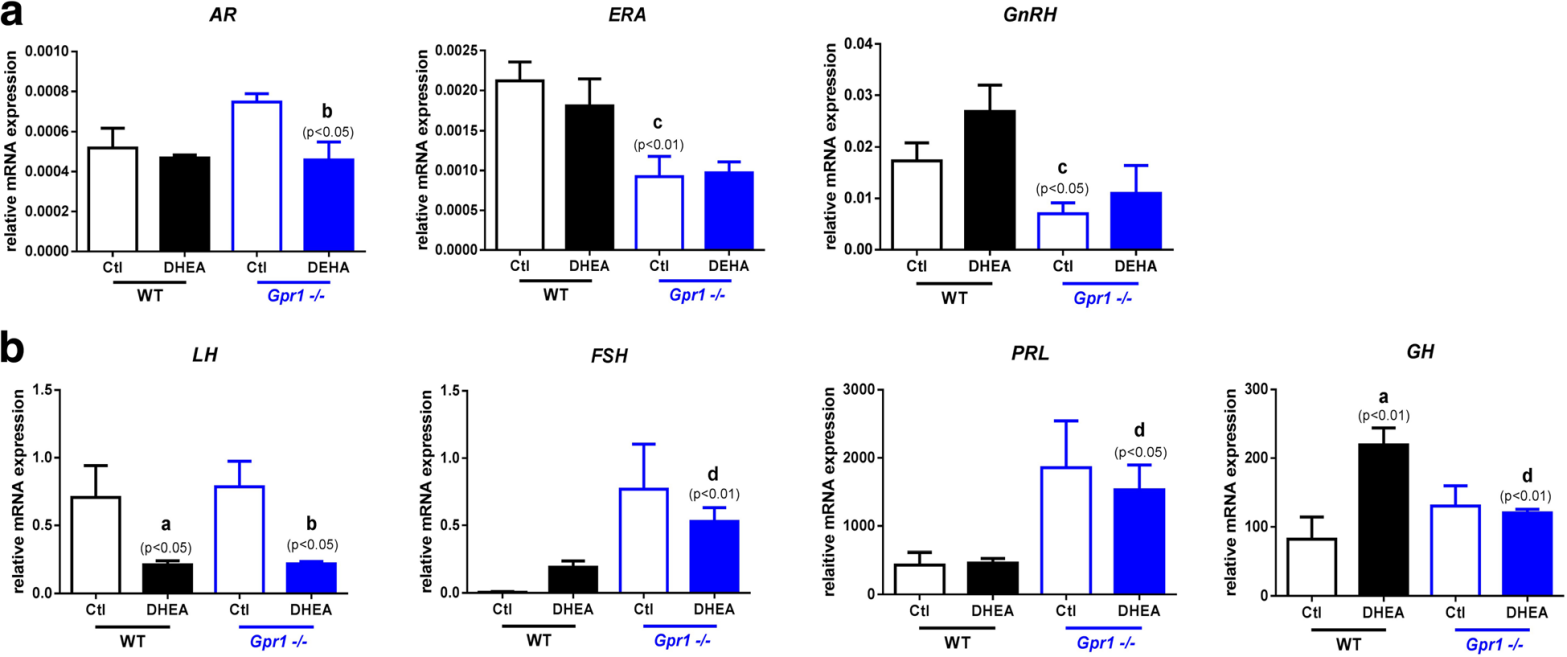
Relative mRNA expression levels in the hypothalamus and pituitary gland in CTL and DHEA-treated wild type and *Gpr1*-null mice (*n* > 6). A Expression of mRNA for steroid receptors and *GnRH* in hypothalamus of CTL and DHEA-treated wild type and *Gpr1*-null mice. B Expression of *LH, FSH, PRL* and *GH* mRNAs in pituitary glands of CTL and DHEA-treated wild type and *Gpr1*-null mice. Data were analyzed using an unpaired Student’s t test. (a) *p*, DHEA-treated vs. control wild type mice; (b) *p*, DHEA-treated vs. control *Gpr1*-null mice; (c) *p,* control *Gpr1*-null mice vs. control wild type mice; (d) *p*, DHEA-treated *Gpr1* null mice vs. DHEA-treated wild type mice

Our data revealed DHEA treatment significantly suppressed AR (androgen receptor) mRNA levels in *Gpr1* knockout mice. ER-A (Estrogen receptor A) and *GnRH* mRNA levels were lower in *Gpr1* knockout mice than in wild type controls (Fig. 2A).

In pituitary glands, *LH* (Luteinizing Hormone) mRNA levels were lower, and *GH* (Growth Hormone) mRNA levels were higher in DHEA-treated control wild type mice. In *Gpr1* knockout mice, the levels of *LH* mRNA were also lower in the DHEA-treated cohort, while the levels of *FSH* (Follicle-Stimulating Hormone) and *PRL* (Prolactin) mRNAs were significantly higher in DHEA-treated *Gpr1* knockout mice than in DHEA-treated wild type mice (Fig. 2B).

Variation in the relative levels of *GH* mRNA was in accordance with variation in bodyweight gain; *Gpr1* deficiency suppressed DHEA-induced increase in relative *GH* mRNA expression. *Gpr1* deficiency also significantly increased the levels of *FSH* and *PRL* mRNAs, whether mice were treated with DHEA or not. Since FSH promotes follicular development and PRL is important for the maintenance and secretory activity of the corpus luteum [32], GPR1 may play a regulatory role in the biosynthesis of ovarian hormones.

### Adipose tissue morphology and expression of mRNAs for steroid receptors

At the end of DHEA treatment, the sizes of scapular, mesenteric, inguinal, and gonadal adipocytes were measured in hematoxylin and eosin (HE)-stained sections from control and DHEA-treated wild type and knockout mice. In each group, the diameters of adipocytes were measured in three randomly selected sections per fat depot per mouse.

**Fig. 3:**
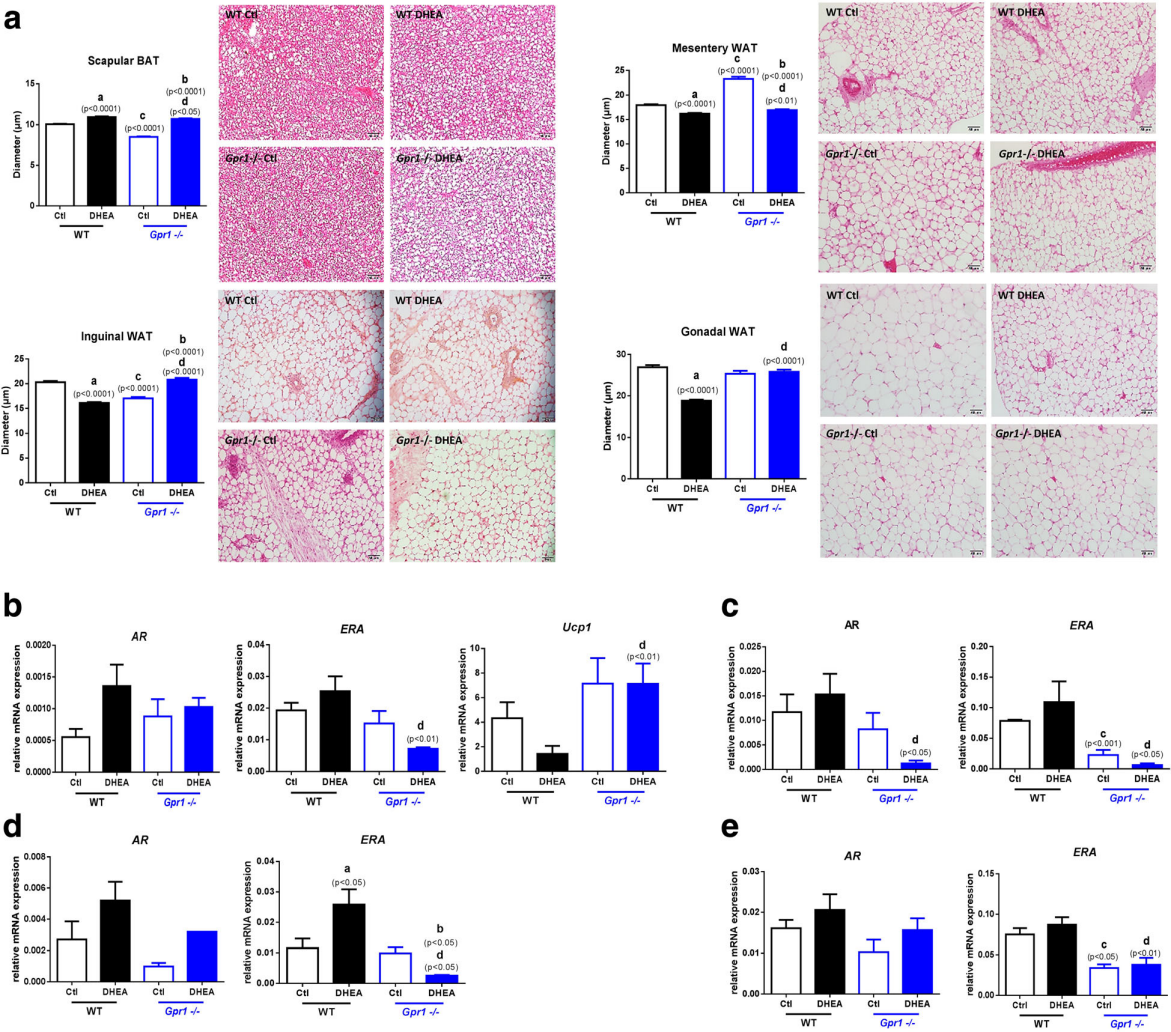
Hematoxylin and eosin staining of brown and white adipocytes and expression of mRNA for steroid receptors in brown and white adipose tissues of CTL and DHEA-treated wild type and *Gpr1*-null mice. A Adipocyte diameters measured in hematoxylin and eosin-stained sections (bar = 50 um) and levels of steroid receptor (B-E) and *Ucp1* mRNAs in scapular brown adipose tissue (B), mesenteric white adipose tissue (C), inguinal white adipose tissue (D), and gonadal white adipose tissue (E). Values for diameters represent means ± SEM (*n* = 6 mice per group). Significance of differences between groups was determined using an unpaired Student’s t test. (a) *p*, DHEA-treated vs. control wild type mice; (b) *p*, DHEA-treated vs. control *Gpr1*-null mice; (c) *p,* control *Gpr1*-null mice vs. control wild type mice; (d) *p*, DHEA-treated *Gpr1* null mice vs. DHEA-treated wild type mice

Our results indicate increased diameters of adipocytes in scapular brown adipose tissue (BAT) but decreased in mesenteric, inguinal, and gonadal adipocytes after DHEA treatment in wild type mice. While in *Gpr1* knockout mice, the diameters were significantly decreased in scapular, but increased in mesenteric, inguinal, and gonadal adipocytes after DHEA treatment when compared with DHEA treated wild type mice. Indicating that loss of *Gpr1* attenuated the effect of DHEA on both brown and white adipose tissues (WAT) (Fig. 3A).

*Gpr1* deficiency significantly elevated *Ucp1* (Uncoupling Protein-1) mRNA abundance, especially with DHEA treatment (Fig. 3B), indicating that GPR1 may play a role in estradiol biosynthesis and thermogenesis in brown adipose tissue.

In mesenteric adipose tissue, the relative levels of *AR* mRNA were significantly lower in the DHEA-treated *Gpr1* knockout mice than in the DHEA-treated wild type mice (Fig. 3C). But for both four kinds of adipose tissues we collected, relative levels of *ERA* mRNA were lower in DHEA-treated *Gpr1* knockout mice than in DHEA-treated wild type mice (Fig. 3B, C, D, E). Which indicating that GPR1 deficiency may attenuated the respond of mesenteric adipose tissue to androgen and the respond of both four adipose tissues to estradiol after DHEA treatment.

### Ovarian features and expression of mRNAs for steroid synthesis enzymes

**Fig. 4:**
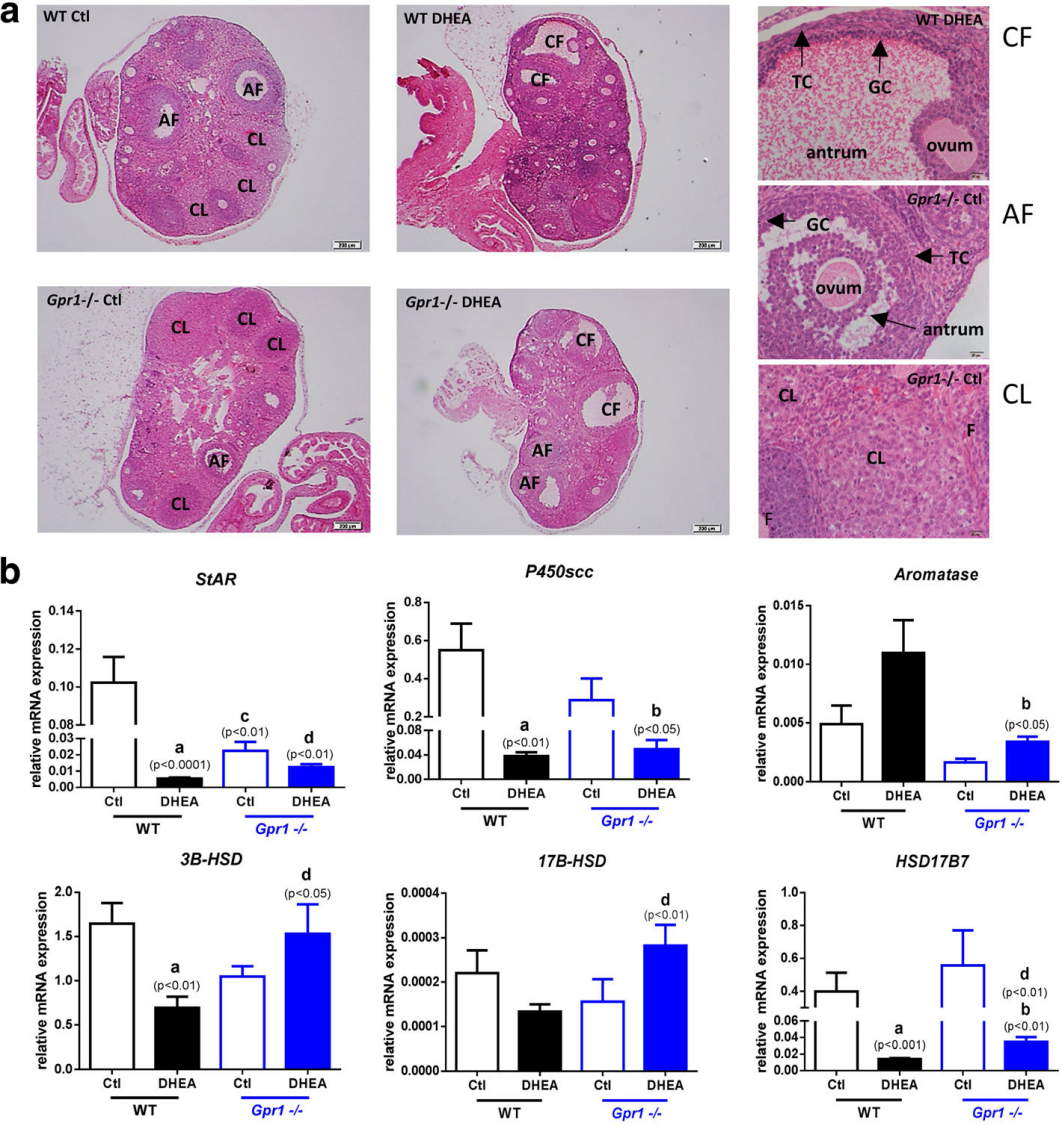
Ovarian features and expression of mRNAs for steroid synthesis enzymes in CTL and DHEA-treated wild type and *Gpr1*-null mice. A Hematoxylin and eosin staining of CTL and DHEA-treated ovaries was examined. The high magnification images on the right show the difference of ovary morphology among various treatment groups. AF, antral follicle. CL, corpus luteum. CF, Cystic follicle. TC, theca cell. GC, granulosa cell. F, follicle. Data represent means ± SEM. B*ar* = 200 μm. B Expression of mRNA for steroid synthesis enzymes in CTL and DHEA-treated wild type and *Gpr1*-null mice. Data were analyzed by an unpaired Student’s t test. (a) *p*, DHEA-treated vs. control wild type mice; (b) *p*, DHEA-treated vs. control *Gpr1*-null mice; (c) *p,* control *Gpr1*-null mice vs. control wild type mice; (d) *p*, DHEA-treated *Gpr1* null mice vs. DHEA-treated wild type mice

To determine the effects of DHEA on ovaries, ovaries were sectioned and stained with hematoxylin and eosin. DHEA treatment of wild type mice resulted in an increase in the number of cystic follicles with degenerated granulosa cells and an absence of corpus luteum. The numbers of antral and pre-antral follicles were increased with arrangement typical of that in polycystic ovaries. The ovaries of *Gpr1*-deficient mice contained follicles at different stages of maturation, with more corpus luteum and mesenchyme. However, ovaries of DHEA-treated *Gpr1*-deficient mice contained still greater numbers of cystic follicles but fewer small follicles than ovaries from DHEA-treated wild type mice, and corpus luteum reoccurred (Fig. 4A).

Then we further investigated mRNA expression for steroid synthesis enzymes in the ovaries of the four experimental groups. The results showed that DHEA treatment significantly reduced the abundance in ovaries of mRNAs for *StAR* (Steroidogenic Acute Regulatory Protein)*, P450scc* (Cytochrome P450), *3B-HSD* (3β-Hydroxysteroid Dehydrogenase) and *HSD17B7* (Hydroxysteroid 17-Beta Dehydrogenase 7) in wild type mice, while the tendency could be all attenuated in *Gpr1*-deficient mice (Fig. 4B).

### Role of mTOR signaling in DHEA-induced, GPR1-mediated ovarian granulosa cell estradiol biosynthesis in vitro

**Fig. 5:**
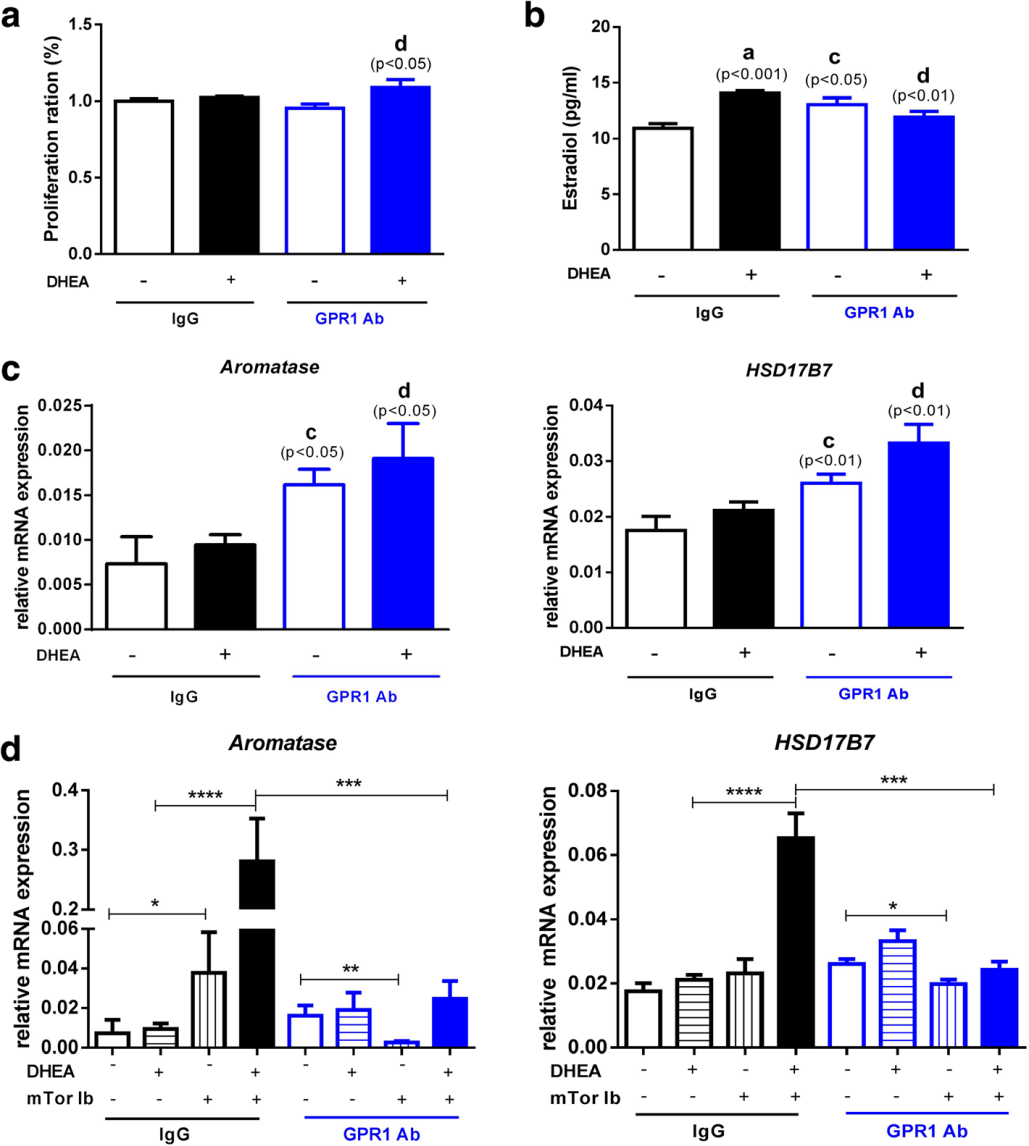
Granulosa cells were isolated after PMSG treatment for 48 h and hCG treatment for 7–12 h and further treated under different conditions for 24 h. A Cell Proliferation under different conditions. B Estradiol levels in culture media from cells under different conditions. C, D Relative mRNA levels for *Aromatase* and *HSD17B7* in granulosa cells under different conditions. Data were analyzed by an unpaired Student’s t test. (a) *p*, DHEA-treated vs. control IgG group; (b) *p*, DHEA-treated vs. control GPR1 antibody group; (c) *p,* control GPR1 antibody group vs. control IgG group; (d) *p*, DHEA-treated GPR1 antibody group vs. DHEA-treated IgG group. (*p < 0.05; **p < 0.01; ***p < 0.001; ****p < 0.0001)

To investigate the mechanism of DHEA-induced, GPR1–mediated estradiol secretion, ovarian granulosa cells were isolated and treated with DHEA or vehicle in the presence of anti-GPR1 antibody or a control antibody, and both cell proliferation and estradiol levels in the conditioned media were measured. We found that the proliferation of granulosa cells was elevated after DHEA treatment when anti-GPR1 antibody was added (Fig. 5A). Estradiol levels were increased in the media from cells treated with either DHEA or GPR1 antibody alone, but GPR1 antibody attenuated the DHEA-induced increase in estradiol (Fig. 5B).

We then isolated cellular RNA and measured the levels of mRNAs for two key enzymes involved in estradiol secretion –*Aromatase* and *HSD17B7* – and found that both were significantly increased in the presence of GPR1 antibody, regardless of whether or not the cells were treated with DHEA. These results are in accordance with measurements of serum estradiol levels (Fig. 5C).

To further explore the pathways involved in DHEA-induced, GPR1-mediated estradiol synthesis, we blocked multiple signaling pathways using specific inhibitors, including the PI3K inhibitor wortmannin (Selleck), the PKC inhibitor Sotrastaurin (Selleck), the AKT inhibitor MK-2206 2HCl (Selleck) (data not shown), and the mTOR inhibitor rapamycin (Selleck) and measured mRNA levels for estradiol synthesis-related enzymes. Inhibition of mTOR with rapamycin caused *Aromatase* mRNA levels to increase in the groups treated with control IgG, with or without DHEA treatment, and *HSD17B7* mRNA levels to increase in cells treated with DHEA and control IgG. In the GPR1 antibody group, the mTOR inhibitor suppressed the expression of *Aromatase* and *HSD17B7* mRNAs. The DHEA-induced increases in *Aromatase* and *HSD17B7* mRNA levels in the IgG group were attenuated by GPR1 antibody (Fig. 5D).

## Discussion

DHEA-induced hyperandrogenemia in mice exhibits some of the phenotypes of PCOS, such as elevated body weight and serum testosterone levels, increased numbers of small follicles, and abnormal steroidogenesis. Since serum Chemerin levels are reported to be higher in women with PCOS [25], and as we previously documented a relationship between the Chemerin receptor CMKLR1 and DHT-induced PCOS in mice, we were curious about whether GPR1, another Chemerin receptor, was also involved in steroid synthesis in hyperandrogenemic mice.

Our examination of systemic indicators revealed that *GPR1* deficiency suppresses DHEA-induced body weight gain, significantly increases serum LDL levels in mice treated with DHEA, and sharply increases serum estradiol levels with or without DHEA treatment. Since obesity is a major characteristic of PCOS [33] and LDL is the major source of cholesterol, a precursor for steroid biosynthesis [34], GPR1 could play a role in both weight change and steroid biosynthesis. However, whether or not change in body weight is caused by changes in fat mass, elevated estradiol levels are caused by the conversion of DHEA or the regulatory effect of GPR1 in the secretion of estrogen during follicular development need further analysis.

To investigate the role of GPR1 in DHEA-induced changes in body weight, samples of brown adipose tissue and of white adipose tissues from three different sites were isolated, and the diameters of adipocytes and alterations in relative gene expression were measured. Our data showed that DHEA treatment increased brown adipocyte diameters but decreased white adipocyte diameters. Decreased white adipocyte diameters, in combination with increased body weight, may result from effects of DHEA on metabolism, such as impairment of fat synthesis and the promotion of fat-free tissue deposition and resting heat production [35]. *Gpr1* deficiency could attenuate DHEA-induced changes in both brown and white adipocytes. Relative expression of *ERA* mRNA was reduced in scapular, mesenteric, inguinal, and gonadal fat in *Gpr1*-deficient mice treated with DHEA. Since adipocytes are the targets of estrogen, estrogen and its receptor could affect adipocyte differentiation, promoting lipolysis and inhibiting lipid synthesis, thereby reducing the deposition of fat [36]. DHEA could be converted to estradiol and activate ERA [37], while *Gpr1* deficiency could attenuate that activation. In addition, the levels of *Ucp1* mRNA in brown adipocytes were significantly elevated in *Gpr1*-deficient mice treated with DHEA, indicating the recovery of heat produced in response to DHEA treatment.

The hypothalamus-pituitary axis is a complex functional system involving both neural and endocrine components. The pituitary secretion of LH and FSH is stimulated by GnRH, further regulating steroid synthesis in ovaries and fat tissues [38].

Steroid synthesis and secretion play an important role in follicular development. To investigate the role of GPR1 in ovarian steroid synthesis, we measured the levels of key limiting steroid synthetic enzymes, including *StAR*, which regulates the transport of cholesterol [39], *P450scc*, a key enzyme in the conversion of cholesterol to pregnenolone [40], *Aromatase*, which catalyzes the conversion of androgens to estrogens [41], *3B-HSD*, which converts pregnenolone to progesterone [42], *17B-HSD* (17β-Hydroxysteroid Dehydrogenase), which converts androgens to estrogens [43], *HSD 17B7*, which is proposed to regulate the balance of E2 and DHT [44], an enzyme that functions as a *17B-HSD* in the biosynthesis of sex steroids [45].

In mice treated with DHEA, we found that *Gpr1* deficiency could attenuate the reduction in levels of steroidogenesis-related enzymes in ovaries. These results indicate that *Gpr1* deficiency may, to some extent, enable the improvement of the abnormal hormone synthesis induced by DHEA. In particular, *Gpr1* deficiency has a tendency to suppress the DHEA-induced elevation in *Aromatase* mRNA expression in ovaries. Since the aromatase inhibitor letrozole has already been shown to be an effective therapeutic drug for PCOS [46, 47], GPR1 may be useful as an alternative or adjunct target for PCOS therapy.

**Fig. 6:**
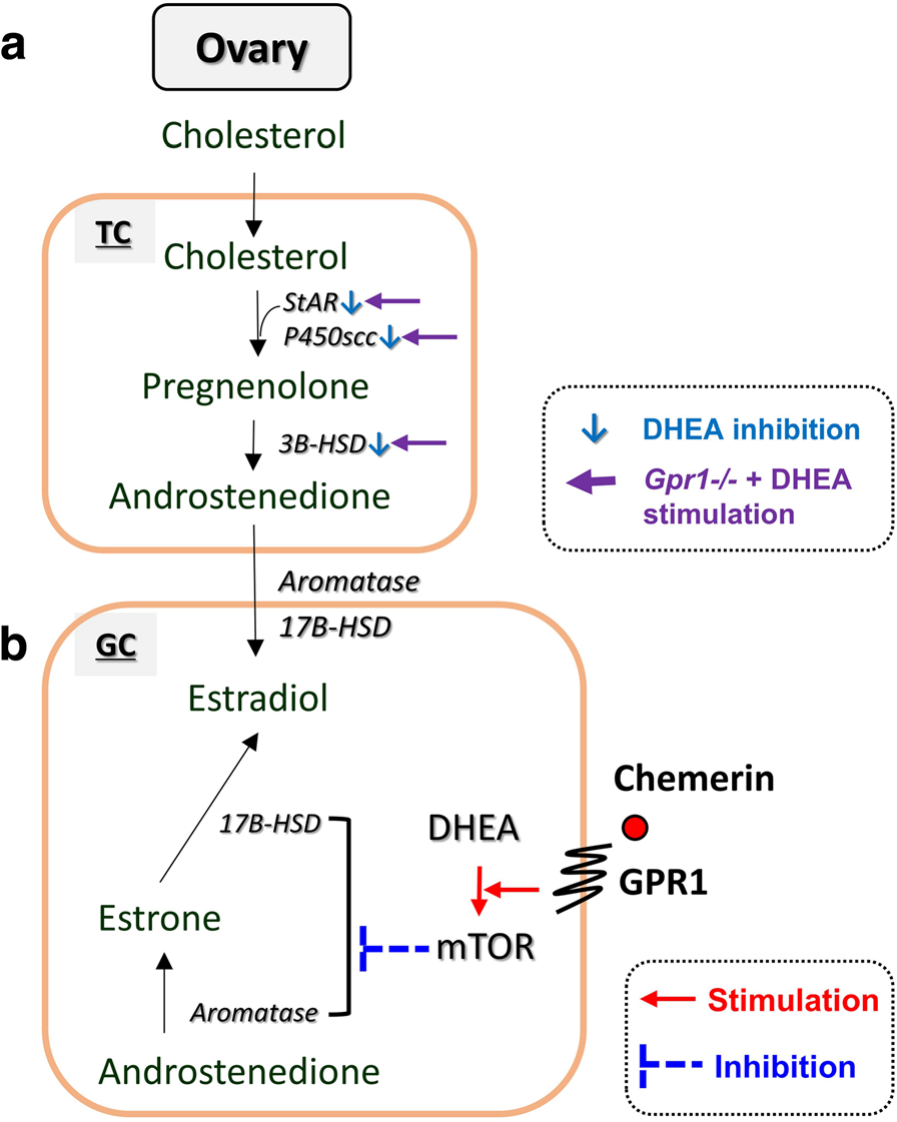
*Gpr1* deficiency attenuates the effect of DHEA and Chemerin/GPR1 signaling modulates estradiol biosynthesis in granulosa cells with DHEA treatment through the mTOR signaling pathway. a DHEA treatment led to decreased ovarian steroidogenesis. *Gpr1* deficiency improved steroidogenesis in ovaries. b Chemerin/GPR1 signaling modulates elevated estradiol secretion induced by DHEA through the mTOR signaling pathway to affect key enzymes involved in estradiol synthesis

To further explore the mechanism of DHEA-induced, GPR1-mediated estradiol secretion, ovarian granulosa cells were isolated and cultured in vitro and the effect of blocking GPR1 function on DHEA induction of estradiol secretion was examined. We found that estradiol levels were elevated after DHEA treatment. Interestingly, even though estradiol levels were also increased by treatment with anti-GPR1 antibody, anti-GPR1antibody and DHEA treatment together led to estradiol levels below that of either treatment alone. From our data, we infer that *Gpr1* deficiency partially attenuates the DHEA-induced increase in estradiol secretion and that it does so through the mTOR signaling pathway to affect key enzymes involved in estradiol synthesis (Fig. 6b).

## Conclusions

In conclusion, our study for the first time demonstrates a regulatory role for the Chemerin-GPR1 pathway in DHEA-induced hyperandrogenism in mice. Since the interaction between organs in the endocrine system is complex, more studies are needed to fully understand the relationships between signals and pathways. However, our findings show that *Gpr1* deficiency can attenuated the negative effects of DHEA, including the increases in body weight and improve steroidogenesis in ovaries (Fig. 6a). Our results indicate that GPR1 could be a new therapeutic target for the treatment of DHEA-induced hyperandrogenism.

## Additional file

Additional file 1: **Figure S1.** Validation for Gpr1 knockout mice. Figure on the left is the reproduction and selection of our Gpr1 knockout mice. Figure on the right is the genotyping of our Gpr1 knockout mice. (TIF 14703 kb)

## Abbreviations

17B-HSD: 17β-Hydroxysteroid Dehydrogenase
3B-HSD: 3β-Hydroxysteroid Dehydrogenase
AR: Androgen Receptor
BAT: Brown Adipose Tissue
CHO: Cholesterol
DHEA: Dehydroepiandrosterone
ER: Estrogen Receptor
FSH: Follicle-Stimulating Hormone
GC: Granulosa Cell
GH: Growth Hormone
GnRH: Gonadotropin Releasing Hormone
HDL: High Density Lipoprotein
HSD17B7: Hydroxysteroid 17-Beta Dehydrogenase 7
LDL: Low Density Lipoprotein
LH: Luteinizing Hormone
P450scc: Cytochrome P450
PCOS: Polycystic Ovary Syndrome
PRL: Prolactin
StAR: Steroidogenic Acute Regulatory Protein
TC: Thecal Cell
TG: Triglyceride
UCP-1: Uncoupling Protein-1
WAT: White Adipose Tissue
