# Supplementary figures and images for: Deficiency of Gpr1 improves steroid hormone abnormality in hyperandrogenized mice
Source: Reprod Biol Endocrinol. 2018 May 24;16:50. doi: 10.1186/s12958-018-0363-9 (PMC5968470; doi:10.1186/s12958-018-0363-9)

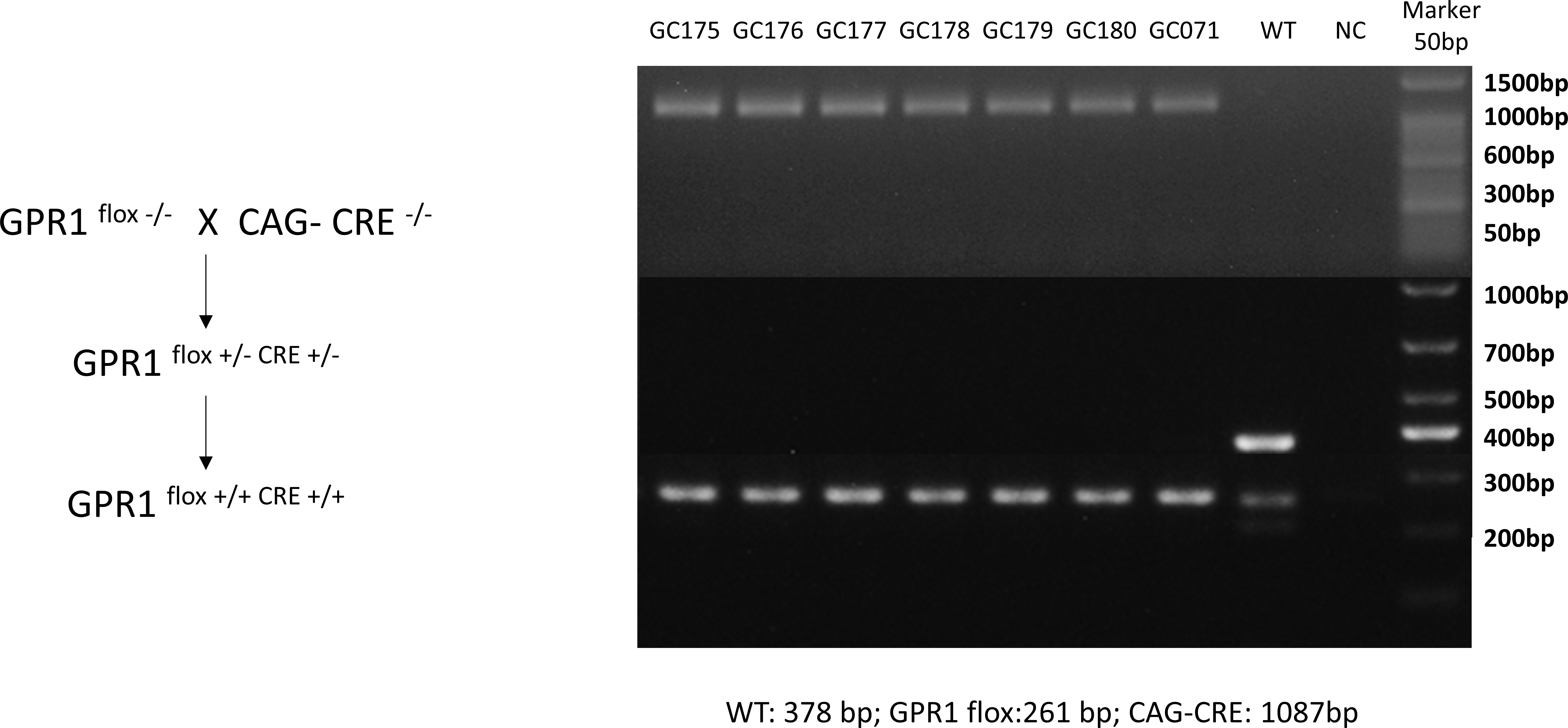

Supplement: Supplementary file 1 — Figure S1. Validation for Gpr1 knockout mice. Figure on the left is the reproduction and selection of our Gpr1 knockout mice. Figure on the right is the genotyping of our Gpr1 knockout mice. (TIF 14703 kb) [file 12958_2018_363_MOESM1_ESM.tif]
